# Multisectoral policy and health systems responses to ageing populations

**DOI:** 10.2471/BLT.25.294250

**Published:** 2026-02-01

**Authors:** Viroj Tangcharoensathien, Jongjit Rittirong, Ritu Sadana, Varalak Srinonprasert, Angkana Lekagul

**Affiliations:** a International Health Policy Program Foundation, Nonthaburi, Thailand.; b Institute of Population and Social Research, Mahidol University, Salaya Campus, 999 Phutthamonthon 4 Road, Salaya, Phutthamonthon, Nakhon Pathom 73170, Thailand.; c Department of Sexual, Reproductive, Maternal, Child and Adolescent Health and Ageing, World Health Organization, Geneva, Switzerland.; d Faculty of Medicine, Siriraj Hospital, Mahidol University, Thailand.

Rapid population ageing, combined with declining fertility rates, is reshaping societies, with far-reaching social, economic and cultural implications. Governments face rising demand for care related to chronic conditions, including dementia and physical impairments, alongside an ageing workforce and increasing dependency ratios that strain health and social systems. These pressures are particularly acute in many low- and middle-income countries, whose populations are ageing before the countries become wealthy and can establish universal health coverage and comprehensive social protection systems.

Effective policy responses require governments to steward coordinated, multisectoral actions. Evidence is needed to guide whole-of-government and whole-of-society approaches that invest in population health^1^ and well-being, raise productivity through education, skills development and technology, and promote lifelong learning. 

The World Health Organization defines healthy ageing as developing and maintaining functional ability that enables well-being in older age.^2^ Operationally, this approach requires policies that build resilience and sustain physical and mental capacities throughout life. When physical and mental declines occur, these need to be mitigated through supportive systems and enabling environments. Valuing and enabling older people’s contributions^3^ is essential to counter fears about labour force contraction. Several countries are already pursuing such strategies, including investments in healthy ageing grounded in a life-course approach.^4^

Translating healthy ageing into policy action requires sustained investments across multiple domains. Priority areas include addressing ageism; creating age-friendly environments; delivering integrated, person-centred care; ensuring access to quality long-term care; and supporting continued employment and social engagement in later life. Well-being must be treated as a core policy outcome: in many settings, loneliness affects more than half of older adults and is strongly associated with poorer health.^5^ Strengthening primary health care, acute and intermediate care, and developing long-term and palliative care services adapted to countries’ socioeconomic and cultural contexts are essential to establishing an effective continuum of care. Crucially, healthy ageing policies must begin early in life through prevention, early detection and treatment of noncommunicable diseases and through actions that reduce long-term disability.

Articles in this theme issue of the *Bulletin of the World Health Organization* expand on these key themes, including how research on the relationship between the exposome, including physical and social environmental exposures, and dementia vulnerability can inform policy and practice to prevent dementia and improve brain health for all people.^6^ Evidence from China shows that dementia-friendly communities integrating nonpharmacological interventions have achieved successful outcomes.^7^

Productivity can increase through better education and health,^8^ and skill development supports employment opportunities for older adults as one means to promote an age-friendly work environment while addressing intergenerational workforce dynamics.

In the context of population decline, improving health and reducing mortality may have a greater impact than ineffective pro-natalist policies.^9^ Although medically assisted reproduction is an essential part of reproductive health care and must be integrated into national health systems, it is costly, inaccessible and only modestly reduces infertility.^10^ When investigating factors influencing fertility decisions, researchers should also examine filial piety, as a social norm shaping fertility and care decisions.^11^ Moreover, health systems responses to an ageing society include ensuring availability of geriatric medicines,^12^ strengthening health information systems for policy decisions^13^ and providing quality long-term care for those in need.^14^
